# The Tin Whistle: A Rare and Serious Cause of Penetrating Oropharyngeal Trauma in Children

**DOI:** 10.1155/2014/562418

**Published:** 2014-08-13

**Authors:** E. C. Francis, K. M. Browne, P. A. Eadie

**Affiliations:** Department of Plastic, Reconstructive and Aesthetic Surgery, Our Lady's Children's Hospital, Crumlin, Dublin 12, Ireland

## Abstract

Impalement injuries of the oral cavity are common in children and the potential for serious complications including internal carotid artery thrombosis can be unnoticed. We present a patient who sustained a penetrating injury in which a “tin whistle” caused herniation of the parotid gland which was not detected on clinical examination. We discuss the challenging clinical examination, the role of investigations, and consequences of these injuries aiming at increasing awareness and optimizing patient management.

## 1. Introduction

Penetrating injuries of the oropharynx and palate are common in children under the age of six [1]. These impalement injuries are especially common amongst toddlers, given their proclivity for mechanical falls while carrying objects in their mouths.

Although the majority of these injuries do not have significant or lasting sequelae, some can have detrimental complications such as involvement of the internal carotid artery with subsequent neurological deficits [2]. Ultimately they can be life-threatening injuries.

Consequently, careful assessment of the patient after the incident is advised. In addition to thorough in-hospital assessment, postdischarge care compromising of parental observation is a key measure to ensure early detection of delayed complications.

## 2. Case History

We present the case of a 20-month-old girl who was referred for review following mechanical fall with a traumatic intraoral laceration.

She had been playing the previous day with her elder sibling with a “tin whistle” in her mouth. She tripped and fell onto her brother sustaining a laceration to her right buccal mucosa. Examination in the emergency department demonstrated a large flap-like laceration involving the buccal mucosa, but it was limited due to pediatric distress and resistance to mouth opening. There were no other associated injuries reported.

**Figure 1:**
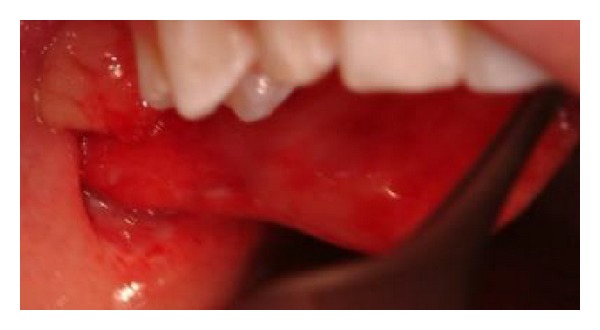
Pedunculated mass arising from the lateral pterygoid region.

Examination under general anaesthesia revealed a 3 × 2 cm soft tissue pedunculated mass arising from the wall of her right buccal mucosa and lateral pterygoid region (Figure 1). It was vascularized with necrotic edges. Its anatomic position near the upper second molar tooth made it clinically consistent with parotid tissue. However, the absence of any significant laceration to the mucosa raised a differential diagnosis of an abnormal growth such as a lymphoma or sarcoma.

**Figure 2:**
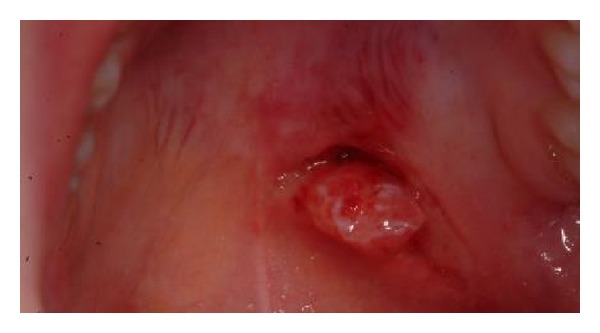
Herniation of the parotid gland and associated buccal fat pad.

**Figure 3:**
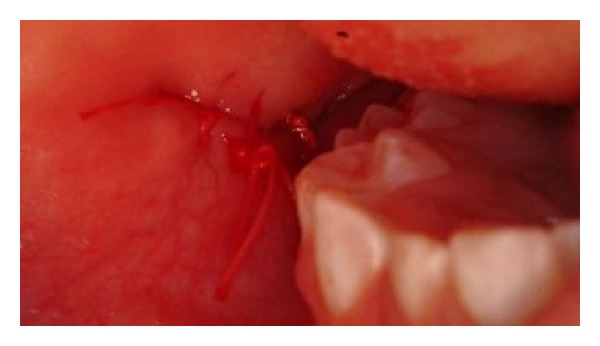
Reduction in herniated contents and closure of wound with absorbable sutures.

Examination revealed the parotid gland and the associated buccal fat pad having herniated through a discrete 1 cm laceration in the wall of the mucosa (Figure 2). The gland and fat pad were atraumatically reduced, the wound edges were sharply and minimally debrided, and the wound was repaired with vicryl rapide sutures (Figure 3).

Postoperatively she was discharged the same day, on a seven-day course of oral antibiotics and her parents were advised regarding the importance of good oral hygiene. She was reviewed in the outpatients six weeks later where her wound had healed without complication.

## 3. Discussion

Children frequently fall with objects in their mouths and injure their oropharynx. These can pose a significant diagnostic challenge to physicians because it is difficult to assess them accurately whilst awake. The majority of these injuries will also tend to typically resolve spontaneously with conservative management without complications [3–6].

However, a small proportion can progress to develop an infection of a deep neck space and/or sustain a carotid artery injury that cause significant morbidity and mortality [7–12].

The approach to blunt oropharyngeal trauma relies on accurate assessment of the wound, use of diagnostic tools as appropriate to the mechanism and history, such as radiology, and/or surgery in specific patients.

In our case, accurate clinical examination while the patient was awake was not possible as, unsurprisingly, the toddler was not cooperative. Ultimately we required an examination under anaesthesia (EUA) to formally assess her injury. There were no concerns regarding lodgment of a foreign body, as the flute was found to be intact after injury and thus plain films were not indicated or required.

Injuries to the oropharynx account for approximately one percent of all pediatric traumas [13]. Commonly reported penetrating objects include toothbrushes, pencils, lollipops, eating utensils, and drinking straws [3, 14].

However to date there has not been a reported case caused by a “tin whistle.” Herniation of the parotid gland has also not been described as a consequence of these injuries. The parotid gland contains several important structures which include the facial nerve, retromandibular vein, external carotid artery, superficial temporal artery, and branches of the great auricular nerve all of which may have been potentially injured causing significant morbidity. In addition to this, there is the accompanying risk of infection causing parotitis.

## 4. Conclusion

Children under the age of six especially toddlers should always be supervised and not be allowed to walk or run with any object in their mouths. Impalement injuries to the oropharynx need to be considered as potentially serious and the foreign body should be removed from the wound only in a controlled environment with careful assessment of any deficit resulting from the trauma. Postinjury monitoring is required even if injury seems to be trivial as complications, especially, neurovascular, may not be clinically apparent immediately.

## Appendix

Tin whistle: Simple six-holed flute metal flute.
